# The prevalence of adult attention-deficit hyperactivity disorder: A global systematic review and meta-analysis

**DOI:** 10.7189/jogh.11.04009

**Published:** 2021-02-11

**Authors:** Peige Song, Mingming Zha, Qingwen Yang, Yan Zhang, Xue Li, Igor Rudan

**Affiliations:** 1 School of Public Health, Zhejiang University School of Medicine, Hangzhou, Zhejiang, China; 2 Medical School Southeast University, Nanjing, Jiangsu, China; 3 Faculty of Life Science and Medicine, Kings College London, London, UK; 4 Centre for Global Health Research, Usher Institute of Population Health Sciences and Informatics, University of Edinburgh, Edinburgh, UK

## Abstract

**Background:**

Adult attention-deficit hyperactivity disorder (ADHD) has recently attracted much attention, however, an up-to-date estimation on the prevalence of adult ADHD is lacking. In this study, we aimed to assess the global prevalence of adult ADHD in the general population through a systematic review and meta-analysis.

**Methods:**

PubMed, Medline, Embase and PsycINFO were searched to identify relevant articles published from January 2000 onwards. Population-based studies that were conducted in the general adult population and quantified the prevalence of adult ADHD were included.

**Results:**

The prevalence of persistent adult ADHD (with a childhood onset) and symptomatic adult ADHD (regardless of a childhood onset) both decreased with advancing age. By adjusting for the global demographic structure in 2020, the prevalence of persistent adult ADHD was 2.58% and that of symptomatic adult ADHD was 6.76%, translating to 139.84 million and 366.33 million affected adults in 2020 globally.

**Conclusions:**

This study provides an up-to-date estimation of the global prevalence of both persistent and symptomatic adult ADHD. A well-defined strategy for diagnosing adult ADHD and large-scale investigations on the epidemiology of adult ADHD are needed.

Attention-deficit hyperactivity disorder (ADHD), a clinically heterogeneous neurodevelopmental syndrome that comprises developmentally inappropriate inattentiveness, hyperactivity and increased impulsivity, is the most common psychiatric disorder in childhood [1]. ADHD significantly impairs multiple aspects of life, leading to educational underachievement, unemployment, unsuccessful marriage and criminality, etc. [2,3]. Moreover, ADHD shows significant correlations with a wide range of comorbid psychiatric disorders, including affective disorders, defiant, antisocial personality disorder, self-harm, substance misuse, placing a considerable burden on society and family [1,4,5].

ADHD has long been conceptualised as a disorder of childhood that gradually diminishes with advancing age during adolescence and young adulthood. However, this assumption was challenged by follow-up studies that observed the persistence of ADHD from childhood into adulthood [6]. Despite the prevailing assumption that adult ADHD and childhood ADHD affect the same group of people and share the same neurodevelopmental aetiology, several prospective longitudinal studies have revealed that more than two-thirds of people with adult ADHD have ever had childhood ADHD [7-9].

Globally, it has been estimated that approximately 5% of children and adolescents are affected by ADHD [10]. Compared with childhood ADHD, adult ADHD is relatively neglected in epidemiological studies, largely due to the absence of well-established and validated diagnostic criteria [6,11,12]. The Diagnostic and Statistical Manual of Mental Disorders (DSM) by the American Psychiatric Association is a widely used approach for diagnosing adult ADHD and requires a childhood-onset [11,12]. Until recently, many attempts have been made to estimate the prevalence of adult ADHD in the general population, either through indirect extrapolating from the prevalence of childhood ADHD with the persistence rate of childhood ADHD to adulthood ADHD, or through direct field investigations [13-15]. Through a systematic review and meta-analysis of six studies published between 1996 and 2005, V Simon and colleagues estimated that the pooled prevalence of adult ADHD was 2.5%. When defining adult ADHD with the DSM-IV criteria, EG Willcutt estimated that the pooled prevalence of adult ADHD was 5.0% based on 11 studies published between 1996 and 2011 [15,16]. Thereafter, more epidemiological investigations on adult ADHD have become available, highlighting the need for an updated estimation of adult ADHD prevalence worldwide.

In this study, we conducted a systematic review of epidemiological studies that reported the prevalence of adult ADHD in the general population. Our objectives were to 1) assess the global prevalence of adult ADHD; 2) explore the potential associated factors of adult ADHD; 3) estimate the global number of people affected by adult ADHD in 2020.

## METHODS

### Search strategy and selection criteria

We conducted this systematic review and meta-analysis in accordance with the Preferred Reporting Items for Systematic Reviews and Meta-Analyses (PRISMA) guidelines [17]. The review protocol was registered online on PROSPERO a prior (CRD42020164602).

On 2 December 2019, two researchers (MZ and QY) independently searched PubMed, Medline, Embase and PsycINFO to identify relevant articles published from 1 January 2000 onwards, that reported the prevalence of adult ADHD in the general population. Search terms related to ADHD (“attention deficit hyperactivity disorder” or “ADHD”), adult (“adult” or “adulthood”), and prevalence (“prevalence” or “epidemiology”) were combined, and the full details of the search strategy are in Table S1 in the **Online Supplementary Document**. The reference lists of relevant systematic reviews were additionally screened to identify eligible publications.

Two researchers (MZ and QY) independently screened titles and abstracts of all retrieved records by literature searches and then reviewed the full-texts of potentially eligible publications. To be included, eligible studies should be primary investigations that were conducted in an adult sample (≥18 years of age). Numerical prevalence estimates of adult ADHD should have been provided, and the definitions and diagnostic approaches of adult ADHD should have been explicitly addressed. Multiple publications from the same investigation were carefully compared, and the one with the largest sample size or the most comprehensive results was kept. Studies that were confined to a subset of adult population with comorbid disorders (people with HIV, diabetes, or other psychiatric disorders, etc.) or in specific settings (prisons, psychiatric units, etc.) were excluded. No language restrictions were applied. Disagreements were resolved by group discussions with a third senior researcher (PS).

### Data extraction and quality assessment

From 12 March 2020 to 15 April 2020, the same two researchers (MZ and QY) independently extracted data using a predefined data collection form. Author, year of publication, title, study location, country, region (as designated by the World Health Organization [WHO] and the World Bank [WB]), year of investigation, study design, sampling strategy, study sample size, age range and female proportion of sample, diagnostic criteria of adult ADHD, and the reported prevalence of adult ADHD (numbers of sample and cases). Where possible, the prevalence rates of adult ADHD were respectively extracted by age group and sex. For articles where the year of investigation was not provided, the mean time lag between investigation and publication (five years, as shown in Table S2 in the **Online Supplementary Document**) was used to impute the year of investigation.

The reporting quality of included articles was examined in reference to the Strengthening the Reporting of Observational Studies in Epidemiology (STROBE) statement [18]. The STROBE-based quality scale consists of five modules: sample population, sample size, participation rate, outcome assessment, and analytical methods. A score of 0-2 was assigned to each module, and the total score represents the overall quality (Table S3 in **Online Supplementary Document**).

### Statistical analysis

As indicated in the previous two systematic reviews of adult ADHD, the definitions of adult ADHD in epidemiological studies varied significantly. Not all the included studies in those meta-analyses required a childhood-onset to define adult ADHD [15,16]. Given the fact that studies that require both a childhood-onset and adult symptoms (persistent adult ADHD) report lower prevalence estimates than those that do not (symptomatic adult ADHD), it is necessary to separately provide prevalence estimates for those two distinct groups. In the present study, the prevalence of persistent adult ADHD and symptomatic adult ADHD was estimated separately.

#### Overall pooled prevalence of adult ADHD

We used a Mantel-Haenszel random-effects meta-analysis to generate the pooled prevalence of persistent adult ADHD and symptomatic adult ADHD. The variance of raw prevalence from all studies was stabilised by using the Freeman-Tukey double arcsine transformation [19]. We used the *I^2^* statistic with ≥50% and the Cochran's Q statistic with a *P* < 0.05 to indicate a significant degree of heterogeneity between studies [20,21]. To examine if the pooled prevalence was affected by extreme contributors, a leave-one-out sensitivity analysis was used for each meta-analysis. Potential publication bias was assessed by visually inspecting the funnel plot, and by Egger's linear regression test and Begg's rank correlation test when ten and more studies were available [22-24].

#### Subgroup meta-analysis of adult ADHD prevalence

Sources of heterogeneity were explored by subgroup meta-analyses for persistent adult ADHD and symptomatic adult ADHD respectively, according to diagnostic tool, DSM version (DSM-IV, DSM-IV-TR, and DSM-V), sex (male and female), setting (urban and rural), investigation period (before 2010, 2010 and later), WHO region (African Region, AFR; Region of the Americas, AMR; South-East Asia Region, SEAR; European Region, EUR; Eastern Mediterranean Region, EMR; and Western Pacific Region,WPR) and WB region (high-income countries, HIC; and low- and middle-income countries, LMIC). As a rule, three and more studies should be available for each subgroup to stabilise the heterogeneity test statistic.

#### Meta-regression of adult ADHD prevalence

Multiple data points were provided within a single study, based on which we established the prevalence of adult ADHD as a function of age. To control the effects of multiple data points from the same study and the same country, a random effect (*u_i_*) was added into the model:


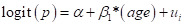
,

Thus, the prevalence of adult ADHD was:


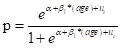
.

Finally, the prevalence of adult ADHD was determined by adjusting for the global demographic structure in 2020, and the number of people affected by adult ADHD in 2020 was calculated by multiplying the age-specific prevalence by the corresponding age-specific population size [25].

Data were analysed using R, version 3.3.0 (R Foundation for Statistical Computing).

## RESULTS

### Study selection and characteristics

**Figure 1:**
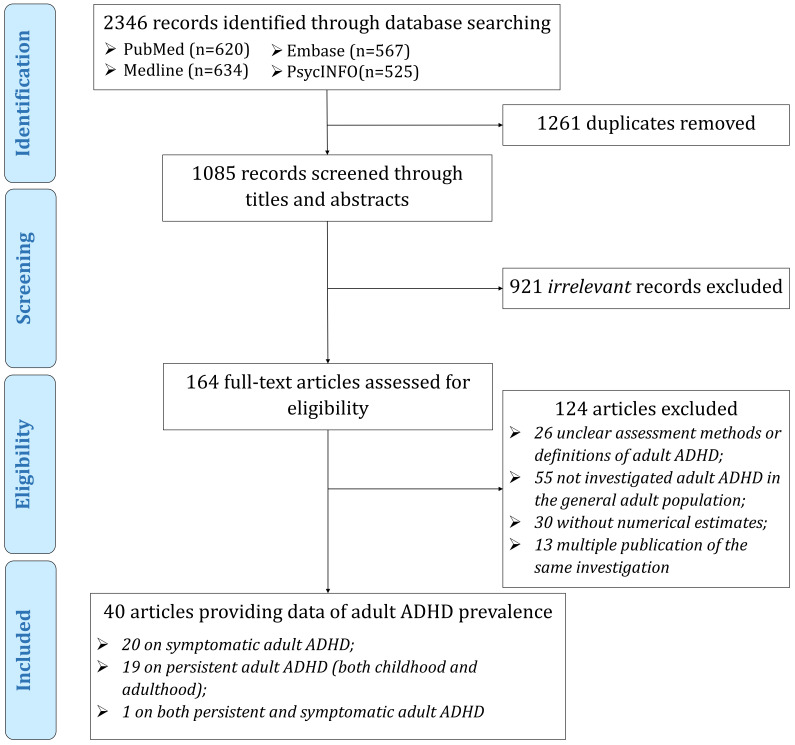
PRISMA flowchart of study selection.

Literature database searches identified 2346 records. After removal of duplicates, 1085 records were screened by title and abstract, resulting in 164 potentially eligible articles for full-text review. Finally, 40 unique articles, covering 30 countries, were included in this systematic review and meta-analysis, among which 20 reported prevalence data on symptomatic adult ADHD, 19 on persistent adult ADHD, and one on both (**Figure 1**). The geographic locations, characteristics, and quality assessments of the included articles are listed in Figure S1, Tables S4-S5 in the **Online Supplementary Document**.

All the diagnosis tools in the 40 articles were based on different versions of DSM. The majority (n = 35, 87.5%) of the 40 articles were published in the last decade (2010-2019), conducted in urban and rural mixed settings (n = 31, 77.5%), or with a quality score of six or above (n = 35, 87.5%). In addition, more than half of the included articles reported the sex-specific prevalence (n = 24, 60.0%), were with a sample size of >1000 (n = 25, 62.5%), or were conducted in HICs (n = 23, 57.5%). Moreover, almost half of the 40 articles provided age-specific prevalence of adult ADHD (n = 17, 42.5%), or were conducted in EUR (n = 17, 42.5%).

### Pooled and stratified prevalence of adult ADHD

As shown in **Table 1**, the prevalence of persistent adult ADHD was reported in 20 articles involving 107 282 individuals. The pooled prevalence by random-effects meta-analysis was 4.61% (95% CI = 3.41-5.99), with high between-study heterogeneity (*I^2^* = 98.8%; *P* < 0.001). By removing a single data point at one time in the sensitivity analysis, the pooled prevalence ranged from 4.22% (95% CI = 3.26-5.29) to 4.88% (95% CI = 3.69-6.22), and no studies were identified as potential outliers. The Egger's test provided evidence of small studies effect (*P* = 0.01) (see Figures S2-S4 in the **Online Supplementary Document** for more details). The prevalence of persistent adult ADHD was also estimated according to diagnostic tool, DSM version, sex, setting, investigation period, WHO region, and WB region, and it was revealed the prevalence of persistent adult ADHD was significantly lower in HICs (3.25%, 95% CI = 2.14-4.57) than in LMICs (8.00%, 95% CI = 3.79-13.57).

**Table 1:** Global prevalence of persistent and symptomatic adult ADHD using random-effects meta-analysis and subgroup meta-analysis

	Number of data points	Number of participants	Number of cases	Prevalence (95% CI)	I^2^	*P*-values			
**Q test**	**Egger's test**	**Begg's test**	**Subgroup difference**
**Persistent adult ADHD**									
Global analysis	20	107 282	2982	4.61 (3.41-5.99)	98.8%	<0.001	0.01	0.19	-
Subgroup analysis									
Diagnostic tool									0.14
ASRS	9	16 021	573	5.95 (3.52-8.95)	97.8%	<0.001	-	-	
Others	11	9 1261	2409	3.71 (2.32-5.39)	99.2%	<0.001	0.17	0.82	
DSM version									0.64
DSM-IV	13	63 959	2288	4.95 (3.49-6.66)	98.5%	<0.001	0.20	0.11	
DSM-IV-TR	4	38 341	510	3.28 (0.99-6.80)	98.4%	<0.001	-	-	
DSM-V	3	4982	184	5.12 (1.20-11.52)	98.1%	<0.001	-	-	
Sex*									0.70
Male	13	37 960	1018	4.99 (3.32-6.96)	97.9%	<0.001	0.04	0.27	
Female	13	44 221	1085	4.49 (2.72-6.66)	98.7%	<0.001	0.04	0.46	
Setting									0.31
Urban	5	8705	176	3.28 (1.6-5.5)	94.6%	<0.001	-	-	
Rural	3	1295	86	11.00 (0.05-35.3)	98.8%	<0.001	-	-	
Investigation period									0.10
Before 2010	15	97 174	2208	3.66 (2.74-4.72)	98.1%	<0.001	0.04	0.59	
2010 and later	5	10 108	774	7.58 (3.18-13.65)	98.9%	<0.001	-	-	
WHO region									0.85
AMR	6	50 426	1256	4.77 (1.74-9.18)	99.6%	<0.001	-	-	
EMR	2	787	67	7.92 (1.13-19.83)	96.0%	<0.001	-	-	
EUR	7	10 350	378	4.64 (2.98-6.62)	94.2%	<0.001	-	-	
WPR	3	7553	144	3.66 (0.79-8.46)	97.5%	<0.001	-	-	
WB region									0.03
HIC	11	59 542	1074	3.25 (2.14-4.57)	97.9%	<0.001	0.00	0.19	
LMIC	7	9574	771	8.00 (3.79-13.57)	98.4%	<0.001	-	-	
**Symptomatic adult ADHD**									
Global analysis	21	50 098	3341	8.83 (7.23-10.57)	97.6%	<0.001	0.00	0.00	-
Subgroup analysis									
Diagnostic tool									0.41
ASRS-6	13	42 304	2499	8.04 (6.61-9.61)	96.6%	<0.001	0.00	0.00	
ASRS-18	7	7217	796	10.1 (5.73-15.53)	97.9%	<0.001	-	-	
DSM version									0.38
DSM-IV	16	45 586	2960	8.36 (6.60-10.30)	97.9%	<0.001	0.02	0.04	
DSM-IV-TR	3	2937	235	10.12 (5.03-16.71)	94.8%	<0.001	-	-	
DSM-V	1	1214	86	7.08 (5.71-8.60)	-	-	-	-	
Sex*									0.85
Male	13	20 742	1284	8.05 (6.1-10.23)	96.2%	<0.001	0.05	0.04	
Female	12	17 040	1085	7.76 (5.79-9.98)	95.7%	<0.001	0.10	0.10	
Setting									0.39
Urban	1	3790	163	4.30 (3.68-4.97)	-	-	-	-	
Rural	2	2170	141	12.19 (0.00-41.23)	99.2%	<0.001	-	-	
Investigation period									0.70
Before 2010	9	18 447	1343	8.47 (6.19-11.07)	96.8%	<0.001	-	-	
2010 and later	12	31 651	1998	9.13 (6.87-11.68)	98.0%	<0.001	0.02	0.02	
WHO region									<0.001
AFR	1	458	42	9.17 (6.69-12.00)	-	-	-	-	
AMR	3	8016	415	6.06 (3.22-9.72)	97.1%	<0.001	-	-	
EMR	2	748	124	16.58 (13.99-19.34)	-	-	-	-	
EUR	10	25 685	1608	7.12 (5.18-9.34)	97.4%	<0.001	0.18	0.13	
SEAR	1	304	78	25.66 (20.90-30.73)	-	-	-	-	
WPR	4	14 887	1074	9.67 (5.72-14.51)	98.3%	<0.001	-	-	
WB region									0.17
HIC	12	40 816	2485	7.66 (6.09-9.39)	97.3%	<0.001	0.01	0.01	
LMIC	9	9282	856	10.68 (6.79-15.32)	97.6%	<0.001	-	-	

CI – confidence interval, WHO – World Health Organization, AFR – African Region, AMR – Region of the Americas, SEAR – South-East Asia Region, EUR – European Region, EMR – Eastern Mediterranean Region, WPR – Western Pacific Region, WB – World Bank, HIC – high-income countries, LMIC – low- and middle-income countries

*The effect of sex was based on articles that provided prevalence data for both males and females.

For symptomatic adult ADHD, the pooled prevalence was 8.83% (95% CI = 7.23-10.57) form 21 included articles involving 50 098 individuals. The between-study heterogeneity was also high (I2 = 97.6%; *P* < 0.001). According to the leave-one-out sensitivity analysis, the pooled prevalence varied from 8.25% (95% CI = 6.75-9.88) to 9.19% (95% CI = 7.52-10.99), no single study significantly influenced the overall pooled prevalence. Potential publication bias was suggested by funnel plot, Egger's test and Begg's test, respectively (Figures S5-S7 in **Online Supplementary Document**). According to the results of sub-group meta-analyses, the prevalence of symptomatic adult ADHD differed significantly across the six WHO regions.

### Age-specific prevalence and the number of cases of adult ADHD in 2020

**Figure 2:**
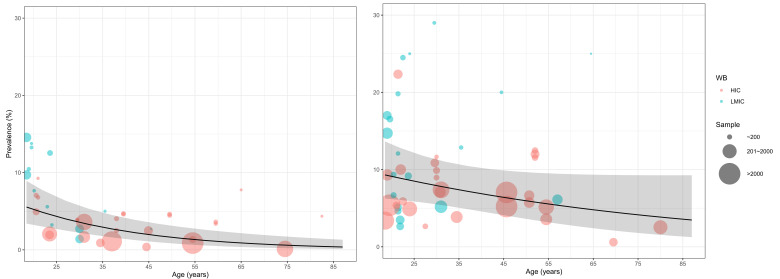
Age- and sex-specific prevalence of adult ADHD based on available data points from the included articles, with 95% confidence intervals.

**Figure 3:**
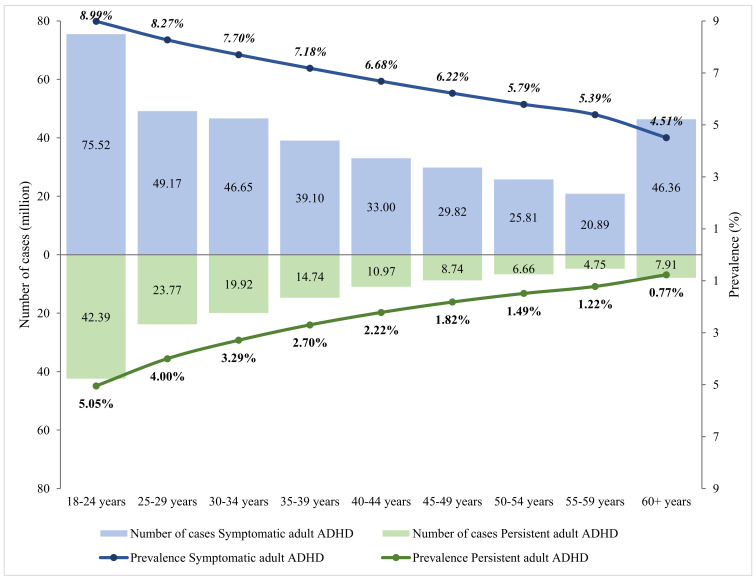
Estimated prevalence and cases of adult ADHD in 2020, by age group.

The relation between age and adult ADHD prevalence was established using multi-level mixed-effects meta-regression (**Figure 2**). By adjusting for the global demographic structure, the prevalence of persistent adult ADHD and symptomatic adult ADHD was 2.58% (95% CI = 1.51-4.45) and 6.76% (95% CI = 4.31-10.61) respectively in 2020 (**Table 2** and **Figure 3**). Generally, the prevalence of persistent and symptomatic adult ADHD both decreased as age increased. The decreasing trend of prevalence with advancing age was more pronounced in persistent adult ADHD, where the prevalence in adults ranged from. 5.05% (95% CI = 3.12-8.07) at 18-24 years of age to 0.77% (95% CI = 0.3-1.96) at ≥60 years. For symptomatic adult ADHD, the estimated prevalence decreased from 8.99% (95% CI = 6.12-13.03) in people aged 18-24 years to 4.51% (95% CI = 2.15-9.32) in those aged 60 years and above. In 2020, a total of 139.84 million (95% CI = 81.64-240.99) people were affected by persistent adult ADHD, and 366.33 million (95% CI = 233.75-574.85) were by symptomatic adult ADHD. The age group that contributed the most adult ADHD cases was 18-24 years.

**Table 2:** Estimated prevalence and cases of adult ADHD in 2020, by age group

Age group (in years)	Persistent adult ADHD		Symptomatic adult ADHD	
**Prevalence (%)**	**Cases (million)**	**Prevalence (%)**	**Cases (million)**
18-24	5.05 (3.12-8.07)	42.39 (26.20-67.73)	8.99 (6.12-13.03)	75.52 (51.40-109.38)
25-29	4.00 (2.49-6.35)	23.77 (14.82-37.77)	8.27 (5.74-11.78)	49.17 (34.11-70.04)
30-34	3.29 (2.02-5.31)	19.92 (12.25-32.15)	7.70 (5.33-11.01)	46.65 (32.28-66.68)
35-39	2.70 (1.61-4.51)	14.74 (8.78-24.55)	7.18 (4.87-10.45)	39.10 (26.55-56.95)
40-44	2.22 (1.26-3.88)	10.97 (6.24-19.14)	6.68 (4.39-10.06)	33.00 (21.66-49.66)
45-49	1.82 (0.98-3.37)	8.74 (4.69-16.16)	6.22 (3.90-9.78)	29.82 (18.70-46.89)
50-54	1.49 (0.75-2.96)	6.66 (3.34-13.18)	5.79 (3.44-9.59)	25.81 (15.32-42.76)
55-59	1.22 (0.57-2.61)	4.75 (2.21-10.11)	5.39 (3.01-9.46)	20.89 (11.66-36.70)
60+	0.77 (0.30-1.96)	7.91 (3.11-20.20)	4.51 (2.15-9.32)	46.36 (22.06-95.80)
Overall (18+)	2.58 (1.51-4.45)	139.84 (81.64-240.99)	6.76 (4.31-10.61)	366.33 (233.75-574.85)

## DISCUSSION

To the best of our knowledge, this systematic review and meta-analysis comprehensively describes the global prevalence of both persistent and symptomatic adult ADHD in the general population based on data published from 2005 to 2019. Our analyses show that the pooled prevalence of persistent adult ADHD was relatively higher in LMICs than in HICs and that of symptomatic adult ADHD varied across WHO regions. We revealed that the prevalence of both persistent and symptomatic adult ADHD decreased as age increased. In 2020, the prevalence of persistent adult ADHD was 2.58%, and that of symptomatic adult ADHD was 6.76%, equivalent to 139.84 million and 366.33 million affected cases globally.

As expected, high levels of heterogeneity of the included studies were noted. Several possible reasons might have contributed to this phenomenon. First, the age of interviewed participants in the included studies was not unified, ranging from young adults to the elderly. Given the fact that the prevalence of adult ADHD decreases with advancing age, as revealed in previous investigations and our meta-regression, it is not surprising to observe such a diversity in the reported prevalence [15,26]. Second, differences in methodologies and case definitions in the reviewed studies might be important sources of heterogeneity, as acknowledged by previous systematic reviews [6,15,16]. In 2009, V Simon and colleagues reported a pooled adult ADHD prevalence of 2.5%, which was coincident with our global prevalence estimation for persistent ADHD in 2020 (2.58%,). However, the study by V Simon and colleagues, have mixed the reported prevalence of persistent ADHD and symptomatic ADHD together, which might lead to difficult-to-interpret findings [15]. To minimise heterogeneity from case definitions, a systematic review by EG Willcutt only included studies that defined adult ADHD based on the DSM-IV approach and revealed a pooled prevalence of 5.0%. Despite the author's efforts, the heterogeneity could not be eliminated [16]. The insufficient understanding of this disorder per se in the adult population and the variety of clinical and pathological features makes the diagnosis of adult ADHD largely dependent on clinical wisdom. Within our persistent adult ADHD group where a childhood-onset is necessary, the age-of-onset greatly differed [27]. For instance, DSM-III and DSM-IV-TR require ADHD symptoms to be present before seven years of age, while DSM-V has broadened the age-of-onset to 12 years. However, according to our subgroup meta-analyses for persistent adult ADHD, the applied DSM version did not seem to explain the heterogeneity. When assessing the presence of adult ADHD symptoms, the WHO Adult ADHD Self-Report Scale (ASRS) was widely used [28-30]. The full ASRS of 18 items collects the frequency of recent adult ADHD symptoms, and the short screener version of ASRS consists of six out of the 18 items. Previous studies have suggested that both ASRS-6 and ASRS-18 were in excellent concordance with clinical diagnoses [29]. In the present study, no significant difference in the prevalence of symptomatic adult ADHD was found in the ASRS-18 and ASRS-6 groups in our subgroup meta-analysis.

Our study distinguishes itself from the abovementioned two systematic reviews by its separate analyses for persistent adult ADHD and symptomatic adult ADHD. Although ADHD has been traditionally under the umbrella of neurodevelopmental disorder, recent evidence has demonstrated that ADHD does not necessarily begin in childhood [7-9]. Holding this conception, several epidemiological investigations have established the prevalence of ADHD solely relying on symptom assessments without additionally requiring a childhood-onset, which constituted the “symptomatic adult ADHD” group in the present study [31-34]. The WHO World Mental Health Surveys (WMHS) across 20 nations have revealed that the prevalence of persistent adult ADHD based on DSM-IV was 2.8% [28]. The estimated prevalence of persistent adult ADHD in our study (2.58%) was similar to that in WMHS, largely supporting our analytic strategy of separating persistent adult ADHD and symptomatic adult ADHD. Other strengths of our study include comprehensive literature searches in multiple data sets and reference lists, which therefore made our study by far a systematic review comprising the most information on the prevalence of adult ADHD. Based on stringent inclusion criteria, we only included studies that were conducted in the general adult population to guarantee the generalisability of our results. Furthermore, we were able to explore a broad range of potential sources of heterogeneity by sub-group meta-analysis, and the prevalence of persistent adult ADHD was found to differ by WB region while that of symptomatic adult ADHD varied by WHO region. For the first time, the age-specific prevalence of persistent adult ADHD and symptomatic adult ADHD was constructed in a systematic review and meta-analysis.

A number of limitations need to be considered in interpreting the results in our study. First, the included studies used different diagnostic criteria and case definitions, were conducted with various study designs, sampling methods and in different study populations, which might have affected the estimated pooled prevalence of both persistent and symptomatic adult ADHD in the present study. Second, the considerable heterogeneity across included studies could not be fully ruled out by a priori selected variables, including diagnostic tool, DSM version, sex, setting, investigation period, WHO region, and WB region. The effects of other potential correlates of adult ADHD, such as ethnicity, were not able to be addressed due to the lack of sufficient information. Third, publication bias might also be a source of heterogeneity, but we did not try to eliminate publication bias in our analyses, because we deemed that an observed prevalence of adult ADHD that substantially differed from previous estimates was likely to have been published.

The lack of internationally acknowledged diagnosis would lead to altered prevalence estimates of adult ADHD across the world. The famous WMHS has investigated the prevalence of DSM-IV-based persistent adult ADHD across 20 countries, while the introduction of DSM-V might lead to a further increase by bringing a rise of age-of-onset from seven years to 12 years and switching the diagnostic emphasis from clinically significant impairments to more lenient symptoms [6,30]. Well-defined diagnostic procedures for adult ADHD should be established by the global research society. Further large-scale international investigations with minimally methodological heterogeneity are still needed to better understand the global epidemiology of both persistent and symptomatic adult ADHD.

## CONCLUSIONS

To conclude, this study reveals that the prevalence of persistent adult ADHD from childhood and that of symptomatic adult ADHD were 2.58% and 6.76% in 2020, representing considerable public health burdens worldwide. Both persistent and symptomatic adult ADHD became less common with advancing age. Development of a universal diagnostic strategy to detect adult ADHD symptoms is greatly needed for both clinical and public health purposes. Large-scale epidemiological investigations with high quality are called for to address the magnitude of adult ADHD across the whole globe.

## Additional material

Online Supplementary Document
