# Supplementary material for: The prevalence of adult attention-deficit hyperactivity disorder: A global systematic review and meta-analysis
Source: J Glob Health. 2021 Feb 11;11:04009. doi: 10.7189/jogh.11.04009 (PMC7916320; doi:10.7189/jogh.11.04009)
Supplement: Online Supplementary Document [file jogh-11-04009-s001.pdf]

Online Supplementary Document

Song et al. The prevalence of adult attention-deficit hyperactivity disorder: a global systematic review and meta-analysis

Table S1. Search strategy to identify studies reporting the prevalence of adult ADHD in the general population

Table S2. The time lag between investigation and publication in the included articles reporting the prevalence of adult ADHD in the general population (n=40)

Table S3. Quality assessment scale for rating the risk of bias

Table S4. Detailed characteristics of the included articles (n=40)

Table S5. Quality scores for assessing the quality of the included articles (n=40)

Figure S1. Location of the included articles reporting the prevalence of persistent adult ADHD and symptomatic adult ADHD

Figure S2. Pooled prevalence of persistent adult ADHD (n=20)

Figure S3. Leave-one-out sensitivity analysis of the influence of single study on the pooled prevalence of persistent adult ADHD

Figure S4. Publication bias of studies on the prevalence of persistent adult ADHD

Figure S5. Pooled prevalence of symptomatic adult ADHD (n=21)

Figure S6. Leave-one-out sensitivity analysis of the influence of single study on the pooled prevalence of symptomatic adult ADHD

Figure S7. Publication bias of studies on the prevalence of symptomatic adult ADHD

This supplementary material has been provided by the authors to give readers additional information about their work.

Table S1. Search strategy to identify studies reporting the prevalence of adult ADHD in the general population

Database	Access date	Search terms
**PubMed**	2nd Dec, 2019	(attention deficit hyperactivity disorder[Title/Abstract] OR ADHD[Title/Abstract]) AND (Adult*[Title/Abstract]) AND (prevalence[Title/Abstract] OR epidemiology[Title/Abstract]) AND ("2000/01/01"[Date - Publication] : "3000"[Date - Publication])<br>Filter: Humans
**MEDLINE (1950-)**	2nd Dec, 2019	1 exp Attention Deficit Disorder with Hyperactivity/<br>2 (attention deficit hyperactivity disorder or ADHD).ab,ti.<br>3 Adult*.ab,ti.<br>4 (prevalen* or epidemiolog*).ab,ti.<br>5 1 or 2<br>6 3 and 4 and 5<br>7 limit 6 to (humans and yr="2000 -Current" and ("all adult (19 plus years)" or "young adult (19 to 24 years)" or "adult (19 to 44 years)" or "young adult and adult (19-24 and 19-44)" or "middle age (45 to 64 years)" or "middle aged (45 plus years)" or "all aged (65 and over)" or "aged (80 and over)") and medline)
**EMBASE (1980-)**	2nd Dec, 2019	1 exp attention deficit disorder/<br>2 (attention deficit hyperactivity disorder or ADHD).ab,ti.<br>3 Adult*.ab,ti.<br>4 (prevalen* or epidemiolog*).ab,ti.<br>5 1 or 2<br>6 3 and 4 and 5<br>7 limit 6 to (human and embase and yr="2000 -Current" and article and journal and (adult <18 to 64 years> or aged <65+ years>))
**PsycINFO**	2nd Dec, 2019	1 exp Attention Deficit Disorder with Hyperactivity/<br>2 (attention deficit hyperactivity disorder or ADHD).ab,ti.<br>3 Adult*.ab,ti.<br>4 (prevalen* or epidemiolog*).ab,ti.<br>5 1 or 2<br>6 3 and 4 and 5<br>7 limit 6 to (human and adulthood <18+ years> and ("0100 journal" or "0110 peer-reviewed journal" or "0120 non-peer-reviewed journal") and journal article and yr="2000 -Current")

Table S2. The time lag between investigation and publication in the included articles reporting the prevalence of adult ADHD in the general population (n=40)

Study ID	Study	Year of publication	Year of investigation	Time-lag (year)
A1[1]	Vigdis Elin Giaever Syrstada, et al.	2020	NA	NA
A2[2]	Kosuke Kajitani, et al.	2019	2017	2
A3[3]	Paulo Mattos, et al.	2018	NA	NA
A4[4]	Flore Moulin, et al.	2018	2001	17
A5[5]	Lucia Romo, et al.	2018	2016	2
A6[6]	Meng Shi, et al.	2018	2016	2
A7_a[7]	Etem Erdal Ersan	2017	NA	NA
A8[8]	John Fayyad, et al.	2017	2006	11
A9[9]	Wakako Ito, et al.	2017	2015	2
A10[10]	Manoj Kumar, et al.	2017	NA	NA
A11[11]	A.Stickleya, et al.	2017	2007	10
A12[12]	E. S. Vitola, et al.	2017	2012	5
A13[13]	ArthurCaye, et al.	2016	2011.5	4.5
A14[14]	Filiz Özdemiroðlu, et al.	2016	2013.5	2.5
A15[15]	Young-sook Kwak, et al.	2015	NA	NA
A16[16]	Terrie E. Moffitt, et al.	2015	1992	23
A17[17]	P.MORTIER, et al.	2015	2013	2
A18[18]	EvelynVingilis, et al.	2015	2012	3
A19[19]	Shahrokh Amiri, et al.	2014	2009	5
A20[20]	Alexandre J. S. MORIN, et al.	2014	2011	3
A21[21]	Estévez N, et al.	2014	2011	3
A22[22]	Tuithof M, et al.	2014	NA	NA
A23[23]	Cortese S, et al.	2013	2004.5	8.5
A24[24]	Jahangard L, et al.	2013	NA	NA
A25[25]	Ashor AW	2012	2011	1
A26[26]	Das D, et al.	2012	2008.5	3.5
A27[27]	de Zwaan M, et al.	2012	2002.5	9.5
A7_b[28]	Kavakci O, et al.	2012	NA	NA
A28[29]	Michielsen M, et al.	2012	2008.5	3.5
A29[30]	de Zwaan M, et al.	2011	2009	2
A30[31]	Park S, et al.	2011	2006.5	4.5
A31[32]	Soysal AS, et al.	2011	2006.5	4.5
A32[33]	Atwoli L, et al.	2010	2009	1
A33[34]	Garnier-Dykstra LM, et al.	2010	2007.5	2.5
A34[35]	Polanczyk G, et al.	2010	2006	4
A7_c[36]	Kilicoglu A, et al.	2009	NA	NA
A35[37]	Pagoto SL, et al.	2009	2002	7
A36[38]	Fayyad J, et al.	2007	2002	5
A37[39]	Faraone SV, et al.	2005	NA	NA
A38[40]	Kooij JJS, et al.	2005	1998	7

Note: NA=not available; The average time-lag between investigation and publication was 5.35 based on 30 articles with available data.

Table S3. Quality assessment scale for rating the risk of bias

Bias type	Low risk (score=2)	Moderate risk (score=1)	High risk (score=0)
Selection (sample population)	1) Sample from the general population, not a select group;<br>2) Consecutive unselected population;<br>3) Rationale for case and control selection explained.	1) Sample selected from large population but selection criteria not defined;<br>2) Sample selection ambiguous but may be representative;<br>3) Rationale for cases and controls not explained;<br>4) Eligibility criteria not explained;<br>5) Analysis to adjust for sampling strategy bias.	1) Highly select population making it difficult to generalise finding;<br>2) Sample selection ambiguous and sample unlikely to be representative.
Selection (sample size)	1) Sample size calculation performed and adequate.	1) Sample size calculation performed and reasons for not meeting sample size given;<br>2) Sample size calculation not performed but all eligible persons studied.	1) Sample size estimation unclear or only sub-sample studied.
Selection (participation rate)	1) High response rate (>85%).	1) Moderate response rate (70-85%).	1) Low response rate (<70%);<br>2) Response rate not reported.
Performance bias (outcome assessment)	1) Diagnosis using consistent criteria and direct examination.	1) Assessment from administrative database or register;<br>2) Assessment from hospital record or interviewer.	1) Assessment from non-validated data or generic estimate from the overall population.
Performance bias (analytical methods to control for bias)	1) Analysis appropriate for the type of sample (subgroup analysis/regression etc.).	1) Analysis does not account for common adjustment.	1) Data confusing.

Table S4. Detailed characteristics of the included articles (n=40)

ID	Country	WHO	WB	Study setting	Origins of sample	Investigation Date	Study design	Sampling Strategy	Diagnostic tools for Adult ADHD	Criteria used for diagnosis	Definition of Adult ADHD	ADHD Category	Sample size	Cases	Age range	Female proportion
A1[1]	Norway	EUR	HIC	The Medical Birth Registry of Norway was used to select a sample from the general population, and covered most of the geographical regions in Norway	Community	NS	Cross-sectional	NS	ASRS-18 items screener	DSM-IV	A positive ADHD screen was defined as having a score of 21 or more on either of the ASRS subscales	Symptomatic adult ADHD	690	75	18-40	0.594203
A2[2]	Japan	WPR	HIC	Kyushu University	University	April 2016-April 2018	Cross-sectional	NS	ASRS-18 items screener	DSM-IV	Each item has a cut-off value (COV) of either 2 (sometimes) or 3 (often) and having four or more items in part A (ASRS-6) above the cut-off is a clinical sign of adult ADHD	Symptomatic adult ADHD	801	179	Mean age: 21.4	0.230961
A3[	Brazil	AMR	UMIC	Universidade Federal do Rio	University	NS	Cross-section	Cluster sampling	ASRS-18 items	DSM-V, previous	Students reporting at	Persistent adult	662	83	Mean	0.581571

ID	Country	WHO	WB	Study setting	Origins of sample	Investigation Date	Study design	Sampling Strategy	Diagnostic tools for Adult ADHD	Criteria used for diagnosis	Definition of Adult ADHD	ADHD Category	Sample size	Cases	Age range	Female proportion
3]				de Janeiro (UFRJ)			al		screener	6 months	least five positive symptoms in the inattention and/or hyperactivity /impulsivity domain were considered "ASRS positive" and were invited to participate in a second stage, which consisted of a semi-structured interview from the ADHD module of the Kiddie Schedule for Affective Disorders and Schizophrenia (K-SADS), in Portuguese,	ADHD			age: 23.6	

ID	Country	WHO	WB	Study setting	Origins of sample	Investigation Date	Study design	Sampling Strategy	Diagnostic tools for Adult ADHD	Criteria used for diagnosis	Definition of Adult ADHD	ADHD Category	Sample size	Cases	Age range	Female proportion
											adapted for adults. In the second stage, occurring on another day and on an individual basis, a final ADHD diagnosis was warranted if the subject met DSM-5 criteria for at least five current inattention and/or hyperactivity /impulsivity symptoms, as well as childhood-onset inattention and/or hyperactivity / impulsivity symptoms occurring in at least two					

ID	Country	WHO	WB	Study setting	Origins of sample	Investigation Date	Study design	Sampling Strategy	Diagnostic tools for Adult ADHD	Criteria used for diagnosis	Definition of Adult ADHD	ADHD Category	Sample size	Cases	Age range	Female proportion
											life domains					
A4[4]	France	EUR	HIC	NS	Community	1991 to 2011	Cross-sectional	NS	ASRS- 6 items screener	DSM-V	Four or more positive responses are considered indicative of clinically significant symptoms (yes vs. no).Kessler's scoring algorithm involves variable cut-points (Items 1-3 use ≥2; Items 4-6 use ≥3) to identify the four or more items	Symptomatic adult ADHD	1214	86	18-37	0.373147
A5[5]	France	EUR	HIC	Rouen University (Normandy) and Paris Nanterre University (Greater Paris area).	University	October 2015 to April 2016	Cross-sectional	NS	ASRS- 6 items screener, WURS-25 items	DSM-IV	Students having a score of 46+ on the WURS and a score of 11+ on ASRS were categorized as having a	Persistent adult ADHD	1517	85	Mean age: 20.6	0.682268

ID	Country	WHO	WB	Study setting	Origins of sample	Investigation Date	Study design	Sampling Strategy	Diagnostic tools for Adult ADHD	Criteria used for diagnosis	Definition of Adult ADHD	ADHD Category	Sample size	Cases	Age range	Female proportion
											potential ADHD					
A6[6]	China	WPR	UMIC	China medical university	University	June 2016	Cross-sectional	Stratified cluster sampling	ASRS- 18 items screener, WURS-25 items	DSM-IV, previous 6 months	Subjects who had a WURS score≥46 and at least one dimension of ASRS score≥17 were classified as symptomatic group.	Persistent adult ADHD	521	44	18-25	0.654511
A7_a[7]	Turkey	EUR	UMIC	The Cumhuriyet University	University	NS	Cross-sectional	Stratified sampling	ASRS- 18 items screener	DSM-IV, previous 6 months	Two standard deviations above ASRS total and sub-scale score averages has been determined as the cutoff point and above this value has been taken as 'group with ADHD symptoms (AADHD)'	Symptomatic adult ADHD	1247	39	18-35	0.577

ID	Country	WHO	WB	Study setting	Origins of sample	Investigation Date	Study design	Sampling Strategy	Diagnostic tools for Adult ADHD	Criteria used for diagnosis	Definition of Adult ADHD	ADHD Category	Sample size	Cases	Age range	Female proportion
A8[8]	20 countries	EUR, EMR, AMR	HIC, LMIC	11 in countries classified by the World Bank (World Bank 2012) as high-income countries (national surveys in Belgium, France, Germany, Italy, Netherlands, Northern Ireland, Poland, Portugal, Spain, and the USA along with a regional survey in Spain [Murcia]), 5 in countries classified as upper-middle-income countries (national surveys in Lebanon and	Community	2001-2012	Cross-sectional	Multistage clustered probability sampling	WHO Composite International Diagnostic Interview (CIDI) version 3.0, Adult ADHD Clinical Diagnostic Scale (ACDS) Version 1.2	DSM-IV, last 12 months	Respondents with symptoms of childhood ADHD were asked whether they still had problems with inattention or impulsivity–hyperactivity and, if so, were asked about impairments due to these symptoms. A probability subsample of 154 such respondents in the US sample was administered blinded clinical follow-up interviews to assess DSM-	Persistent adult ADHD	26744	749	18-44	0.570334

ID	Country	WHO	WB	Study setting	Origins of sample	Investigation Date	Study design	Sampling Strategy	Diagnostic tools for Adult ADHD	Criteria used for diagnosis	Definition of Adult ADHD	ADHD Category	Sample size	Cases	Age range	Female proportion
				Romania, a survey in all urbanized areas of Mexico, and regional surveys in Brazil [Sao Paulo] and Colombia [Medellin]), and 4 in countries classified as low-/lower-middle-income (national surveys in Colombia and Iraq, a survey in all urbanized areas of Peru, and a regional survey in the People's Republic of China [Shenzhen]).							IV adult ADHD using the validated form of the Adult ADHD Clinical Diagnostic Scale (ACDS) Version 1.2					
A 9[	Japan	WPR	HIC	Japan	Community	Feb 2015	Cross-section	Random sampling	ASRS-v1.1-6 items	DSM-IV, last 6	Participants in this study	Symptomatic	9822	602	20-69	0.5

ID	Country	WHO	WB	Study setting	Origins of sample	Investigation Date	Study design	Sampling Strategy	Diagnostic tools for Adult ADHD	Criteria used for diagnosis	Definition of Adult ADHD	ADHD Category	Sample size	Cases	Age range	Female proportion
9]							al		screener	months	were classified as “possible ADHD” if their ASRS score was 14 and “non-ADHD” if the ASRS score was <14	adult ADHD				
A10[10]	India	SEAR	LMIC	A village of south India	Community	NS	Cross-sectional	Cluster sampling	ASRS- 6 items screener	DSM-IV	A cut off score of 14 on the six-item version were taken as suggestive of ADHD.	Symptomatic adult ADHD	304	78	19+	NS
A11[11]	United Kingdom	EUR	HIC	England	Community	October 2006 to December 2007	Cross-sectional	Multistage stratified probability sampling	ASRS- 6 items screener	DSM-IV, previous 6 months	A score of 14 and above being used to signify the possible presence of ADHD	Symptomatic adult ADHD	7268	387	18-95	0.569895
A12[12]	Brazil	AMR	UMIC	Pelotas	Community	2012	Cohort	Stratified sampling	ASRS- 6 items screener	DSM-V, last 6 months	At least 5/9 inattention and/or 5/9 hyperactivity symptoms + symptom onset before	Persistent adult ADHD	3369	70	30	0.481745

ID	Country	WHO	WB	Study setting	Origins of sample	Investigation Date	Study design	Sampling Strategy	Diagnostic tools for Adult ADHD	Criteria used for diagnosis	Definition of Adult ADHD	ADHD Category	Sample size	Cases	Age range	Female proportion
											age 12 + symptoms in more than one setting + moderate or severe impairment related to ADHD symptoms					
A13[13]	Brazil	AMR	UMIC	Pelotas	Community	2011-2012	Cohort	NS	A screening instrument (hyperactivity subscale of the Strength and Difficulties Questionnaire) calibrated for a DSM-IV ADHD diagnosis based on clinical interviews with parents using the Developme	DSM-IV	The optimal cutoff for ADHD was estimated to be 8 or more points on the SDQ hyperactivity scale as rated by parents	Persistent adult ADHD	4039	492	18-19	0.510275

ID	Country	WHO	WB	Study setting	Origins of sample	Investigation Date	Study design	Sampling Strategy	Diagnostic tools for Adult ADHD	Criteria used for diagnosis	Definition of Adult ADHD	ADHD Category	Sample size	Cases	Age range	Female proportion
									nt and Well-Being Assessment							
A14[14]	Turkey	EUR	UMIC	Adnan Menderes University Faculty of Medicine	University	2013-2014	Cross-sectional	NS	Diagnostic interviews, Adult ADD/ADHD Scale	DSM-IV	In the scoring, it is accepted that there is a lack of attention (DE) in the person who received 2 or 3 answers to at least six of the 9 questions in the first section. In the second part, if at least six of the 9 questions were received in 2 or 3 answers, this person has excessive mobility / impulsivity (AH). By gathering the answers	Symptomatic adult ADHD	577	46	Mean age: 20.42	0.518198

ID	Country	WHO	WB	Study setting	Origins of sample	Investigation Date	Study design	Sampling Strategy	Diagnostic tools for Adult ADHD	Criteria used for diagnosis	Definition of Adult ADHD	ADHD Category	Sample size	Cases	Age range	Female proportion
											given to the questions in the third section, there are points related to ADD / ADHD					
A15[15]	Korea	WPR	HIC	12 universities of Korea	University	NS	Cross-sectional	Stratified cluster sampling	ASRS-v1.1-6 items screener	DSM-IV, past 6 months	Presence of ADHD symptoms in adults was represented by scores of 14 and above	Symptomatic adult ADHD	2172	164	18+	0.482044
A16[16]	New Zealand	WPR	HIC	Dunedin	Community	1972-2011	Cohort	NS	Diagnostic Interview Schedule for Children-Child Version, Private structured diagnostic interviews	DSM-III, DSM-5, past 12 months	Symptoms were ascertained using the Diagnostic Interview Schedule for Children-Child Version at ages 11 and 13 by a child psychiatrist and at age 15 by trained interviewers. Symptoms	Persistent adult ADHD	951	31	38	0.504732

ID	Country	WHO	WB	Study setting	Origins of sample	Investigation Date	Study design	Sampling Strategy	Diagnostic tools for Adult ADHD	Criteria used for diagnosis	Definition of Adult ADHD	ADHD Category	Sample size	Cases	Age range	Female proportion
											were ascertained when participants were age 38 through private structured diagnostic interviews by trained interviewers with mental-health-related tertiary qualifications and clinical experience					
A17[17]	Belgium	EUR	HIC	Belgium	University	September 2012 to June 2014	Cross-sectional	NS	ASRS- 6 items screener	DSM-IV, past 6 months	The cut-off score of 14 was therefore seen as a possible ADHD diagnosis, 18 as a likely ADHD diagnosis	Symptomatic adult ADHD	4921	265	18-20+	0.55192
A1	Canada	AMR	HIC	Ontario	Community	January 2011	Cross-section	Two-stage probabilit	ASRS-v1.1-6 items	DSM-IV	Positive ADHD	Symptomatic	3929	133	18+	0.534233

ID	Country	WHO	WB	Study setting	Origins of sample	Investigation Date	Study design	Sampling Strategy	Diagnostic tools for Adult ADHD	Criteria used for diagnosis	Definition of Adult ADHD	ADHD Category	Sample size	Cases	Age range	Female proportion
8[18]						to December 2012	al	y sampling	screener		symptoms screen is a total score greater than 13	adult ADHD				
A19[19]	Iran	EMR	UMIC	Urban Tabriz, North-West of Iran	Community	2009	Cross-sectional	Two-stage cluster sampling	Conner's Adult ADHD Rating Scale (CAARS), K-SADS questionnaire, psychiatric interview	DSM-IV-TR criteria and Wender Utah	The subjects with ADHD index higher than 70 in the self-report questionnaire of Conner's Adult ADHD Rating Scale were initially evaluated for childhood ADHD symptoms; and if positive, the ADHD portion of K-SADS questionnaire about childhood was fulfilled; and if certified by one more	Persistent adult ADHD	400	15	18-50	0.5

ID	Country	WHO	WB	Study setting	Origins of sample	Investigation Date	Study design	Sampling Strategy	Diagnostic tools for Adult ADHD	Criteria used for diagnosis	Definition of Adult ADHD	ADHD Category	Sample size	Cases	Age range	Female proportion
											family member knowing about his/her childhood, the subject was selected. Then, the psychiatric interview was performed for all selected individuals according to the DSM-IV-TR criteria and Wender Utah; and those who had definite symptoms were diagnosed to have adult ADHD					
A20[2	France	EUR	HIC	20 kindergarten schools, 30 primary	Community	Spring 2010 to 2011	Cross-sectional	NS	ASRS - 6 items screener	DSM-IV	Those who received a total score higher than	Symptomatic adult ADHD	1171	131	20-64	0.564475

ID	Country	WHO	WB	Study setting	Origins of sample	Investigation Date	Study design	Sampling Strategy	Diagnostic tools for Adult ADHD	Criteria used for diagnosis	Definition of Adult ADHD	ADHD Category	Sample size	Cases	Age range	Female proportion
0]				schools , and 14 secondary schools from Southern France							10 are considered as very likely to have ADHD					
A21[21]	Switzerland	EUR	HIC	Three of a total of six centres that recruit men for military service, covering 21 of 26 Swiss cantons (including all French-speaking cantons).	Community	August 2010-November 2011	Cohort	NS	ASRS-v1.1-6 items screener	DSM-IV, last 12 months	The answers then were dichotomized into the variable "no ADHD" (scores 0–13) and "ADHD" (scores 14–24)	Symptomatic adult ADHD	5656	226	17-28	0
A22[22]	Netherlands	EUR	HIC	NS	Community	NS	Cross-sectional	Stratified, multi-stage, random sampling	Composite International Diagnostic Interview (CIDI) version 3.0	DSM-IV	CIDI 3.0 asks about symptoms of ADHD in childhood and about the burden the respondent has experienced. The answers reveal whether the	Persistent adult ADHD	3305	55	18-44	0.4965

ID	Country	WHO	WB	Study setting	Origins of sample	Investigation Date	Study design	Sampling Strategy	Diagnostic tools for Adult ADHD	Criteria used for diagnosis	Definition of Adult ADHD	ADHD Category	Sample size	Cases	Age range	Female proportion
											person meets the DSM-IV criteria for ADHD in childhood and early adolescence. In addition, those with ADHD in childhood were asked if the ADHD symptoms were still present in the past year.					
A23[23]	USA	AMR	HIC	Households and group quarters (such as college halls of residents, barracks, residential treatment centres) in the USA, including Alaska and Hawaii	Community	2004-2005	Cohort	NS	Self-reported screening for ADHD in adulthood/ adolescence	DSM-IV-TR, last 6 months	For the diagnosis of ADHD, six or more inattention and/or impulsive–hyperactive symptoms had to be present for at least 6 months (criterion A),	Persistent adult ADHD	34653	340	18+	0.521

ID	Country	WHO	WB	Study setting	Origins of sample	Investigation Date	Study design	Sampling Strategy	Diagnostic tools for Adult ADHD	Criteria used for diagnosis	Definition of Adult ADHD	ADHD Category	Sample size	Cases	Age range	Female proportion
											be associated with impairment in two or more settings (criterion C) and interfere significantly with social, school or work functioning (criterion D). Symptoms had to be present before the age of 12. Persistent ADHD was defined by the current presence of the aforementioned criteria at Wave 2					
A24[2	Iran	EMR	UMIC	Hamadan University of Medical Sciences	University	NS	Cross-sectional	NS	ASRS-v1.1-6 items screener (and	DSM-IV-TR	ASRS V1.1 symptom checklist requires that	Both symptomatic and	387	64 (adulthood)	Mean age: 19.	0.66

ID	Country	WHO	WB	Study setting	Origins of sample	Investigation Date	Study design	Sampling Strategy	Diagnostic tools for Adult ADHD	Criteria used for diagnosis	Definition of Adult ADHD	ADHD Category	Sample size	Cases	Age range	Female proportion
4]				(Hamadan, Islamic Republic of Iran)					Wender Utah Rating Scale [WURS])		the first three items be present at least "sometimes" and the next three to be present "often" to qualify for a screen positive status of ADHD	persistent adult ADHD		and 52 (both childhood and adulthood)	6	
A25[25]	Iraq	EMR	UMIC	Department of Pharmacology, University of Al-Mustansiriya	University	March 2011 to June 2011	Cross-sectional	Stratified cluster sampling	ASRS- 6 items screener	DSM, last 6 months	Participants for whom total scores for these 6 items were 14 or higher, were considered ADHD positive	Symptomatic adult ADHD	361	60	18-25	0.412742
A26[26]	Australia	WPR	HIC	The city of Canberra and the adjacent town of Queanbeyan in south-eastern Australia	Community	2007-2010	Cohort	NS	ASRS- 6 items screener	DSM-IV-TR, last 6 months	Scores 14–24 being indicative of possible ADHD	Symptomatic adult ADHD	2092	129	47-54	0.525335

ID	Country	WHO	WB	Study setting	Origins of sample	Investigation Date	Study design	Sampling Strategy	Diagnostic tools for Adult ADHD	Criteria used for diagnosis	Definition of Adult ADHD	ADHD Category	Sample size	Cases	Age range	Female proportion
A27[27]	Germany	EUR	HIC	Representative areas of Germany	Community	2002-2003	Cross-sectional	Random sampling	Wender Utah Rating Scale (WURS-k), ADHD self-rating scale (ADHD-SR)	DSM-IV-TR	A cut off score of >=30 to indicate the presence of a diagnosis of ADHD in childhood. The recommended cut-off score of >=15 was used to indicate that participants met criteria for adult ADHD. Only participants who fulfilled both the WURS-k criteria and the ADHD-SR criteria were diagnosed as cases of adult ADHD	Persistent adult ADHD	1655	78	18-64	0.551057
A7_b[2	Turkey	EUR	UMIC	Cumhuriyet University (CU), located in central	University	NS	Cross-sectional	Simple random sampling	ASRS- 18 items screener	DSM-IV	1.5 SD abo ve the mean-score of 40	Symptomatic adult ADHD	980	48	17-44	0.441

ID	Country	WHO	WB	Study setting	Origins of sample	Investigation Date	Study design	Sampling Strategy	Diagnostic tools for Adult ADHD	Criteria used for diagnosis	Definition of Adult ADHD	ADHD Category	Sample size	Cases	Age range	Female proportion
8]				Anatolia in Turkey												
A28[29]	Netherlands	EUR	HIC	Population registries of 11 municipalities in 3 geographical areas of The Netherlands	Community	2008-2009	Cohort	Two-phase non-proportional stratified random sampling	Attention-deficit hyperactivity disorder was screened using a questionnaire developed by Barkley et al (Barkley RA, Murphy KR, Fischer M. ADHD in Adults: What the Science Says. Guilford, 2007); To diagnose ADHD, the Diagnostic Interview for ADHD in Adults, second	DSM-IV-TR, last 6 months	Symptomatic ADHD required the cut-off score of four symptoms or more of either inattention and/or hyperactivity –impulsivity during the 6 months prior to the interview and >=6 symptoms of inattention and/or hyperactivity –impulsivity in childhood	Persistent adult ADHD	231	23	60-94	0.59

ID	Country	WHO	WB	Study setting	Origins of sample	Investigation Date	Study design	Sampling Strategy	Diagnostic tools for Adult ADHD	Criteria used for diagnosis	Definition of Adult ADHD	ADHD Category	Sample size	Cases	Age range	Female proportion
									edition, (Diagnostisch Interview Voor ADHD bij volwassenen, DIVA 2.0) was used							
A29[30]	Germany	EUR	HIC	The area of Germany was separated into 258 sample areas representing the different regions of the country.	Community	November 27 2009-December 16, 2009	Cross-sectional	NS	Wender Utah Rating Scale (WURS-k), ADHD self-rating scale (ADHD-SR)	DSM-IV	A cut off score of >=30 to indicate the presence of a diagnosis of ADHD in childhood. The recommended cut-off score of >=15 was used to indicate that participants met criteria for adult ADHD. Only participants who fulfilled both the WURS-k criteria and the ADHD-SR	Persistent adult ADHD	1633	77	18-64	0.536

ID	Country	WHO	WB	Study setting	Origins of sample	Investigation Date	Study design	Sampling Strategy	Diagnostic tools for Adult ADHD	Criteria used for diagnosis	Definition of Adult ADHD	ADHD Category	Sample size	Cases	Age range	Female proportion
											criteria were diagnosed as probable cases of adult ADHD					
A30[31]	Korea	WPR	HIC	Korea	Community	2006-2007	Cross-sectional	Stratified, multi-stage, cluster sampling	ASRS-v1.1-6 items screener	DSM-IV, last 6 months	A dichotomous version of the six-question ASRS-v1.1 Screener, wherein scores of 4 and above were considered consistent with adult ADHD. In addition to obtaining self-reported symptom frequencies, the interviewer confirmed the persistence of at least one symptom from	Persistent adult ADHD	6081	69	18-59	0.495

ID	Country	WHO	WB	Study setting	Origins of sample	Investigation Date	Study design	Sampling Strategy	Diagnostic tools for Adult ADHD	Criteria used for diagnosis	Definition of Adult ADHD	ADHD Category	Sample size	Cases	Age range	Female proportion
											childhood					
A31[32]	Turkey	EUR	UMIC	Gazi University Department of Nursing	University	2006-2007	Cross-sectional	Cluster sampling	Wender Utah Rating Scale (WURS), ADHD scale	DSM-IV	WURS >=36; If 2 or 3 answers are given to at least six of the 9 questions in the first part of the scoring, attention deficit disorder is diagnosed.	Persistent adult ADHD	196	15	Mean age: 20.16	NS
A32[33]	Kenya	AFR	LMIC	The study was carried out at the Town Campus of Moi University. The campus is located in Eldoret town.	University	March 2009 to October 2009	Cohort	Cluster sampling	ASRS-v1.1-18 items screener	DSM-IV-TR, last 6 months	To meet the criteria for possible ADHD, at least six of either the inattentive symptoms (Questions 1-4 and 7-11 on the ASRS checklist) or the hyperactivity /impulsivity symptoms (Questions 5,	Symptomatic adult ADHD	458	42	17-46	0.485

ID	Country	WHO	WB	Study setting	Origins of sample	Investigation Date	Study design	Sampling Strategy	Diagnostic tools for Adult ADHD	Criteria used for diagnosis	Definition of Adult ADHD	ADHD Category	Sample size	Cases	Age range	Female proportion
											6 and 12-18 on the ASRS checklist) had to be positive on the full ASRS symptom checklist					
A33[34]	USA	AMR	HIC	A large public mid-Atlantic university	University	2007-2008	Cohort	Cluster sampling	ASRS- 18 items screener	DSM-IV	Scores of 0–3 and 4–8 denoted “low” and “moderate” ADHD symptoms, respectively, and a score of 9 or higher fell into the “clinical” range, indicating the participant might benefit from a clinical assessment for ADHD	Symptomatic adult ADHD	1080	108	21-23	0.538
A34[	Brazil	AMR	UMIC	143 counties selected from Brazil	Community	November 2005-	Cross-sectional	Three-stage cluster	ASRS-6 items screener	DSM-IV	A positive screening in the ASRS	Symptomatic adult	3007	174	18+	NS

ID	Country	WHO	WB	Study setting	Origins of sample	Investigation Date	Study design	Sampling Strategy	Diagnostic tools for Adult ADHD	Criteria used for diagnosis	Definition of Adult ADHD	ADHD Category	Sample size	Cases	Age range	Female proportion
35]						April 2006		sampling			Screener was defined as a score of 14 or higher	ADHD				
A7_c[36]	Turkey	EUR	UMIC	Dumlupinar university	University	NS	Cross-sectional	Cluster sampling	Adult ADHD Scale- 18 items screener	DSM-IV	In general, those with a score below 20 show low levels of ADHD, those with a score between 20-59 indicate moderate levels of ADHD, and those with a score above 59 indicate high levels of ADHD	Symptomatic adult ADHD	1961	305	Mean age: 18.97	0.634
A35[37]	USA	AMR	HIC	NS	Community	2001-2003	Cross-sectional	Four-stage probability sampling	Adult ADHD Clinical Diagnostic Scale (ACDS)	DSM-IV, last 6 months	Respondents were diagnosed with adult ADHD if they endorsed six or more symptoms of either inattention or	Persistent adult ADHD	6737	243	18-44	0.516

ID	Country	WHO	WB	Study setting	Origins of sample	Investigation Date	Study design	Sampling Strategy	Diagnostic tools for Adult ADHD	Criteria used for diagnosis	Definition of Adult ADHD	ADHD Category	Sample size	Cases	Age range	Female proportion
											hyperactivity within the last six months (DSM-IV Criterion A), two or more Criterion A symptoms before age seven (Criterion B), impairment in two or more areas of living within the past six months (Criterion C), and clinically significant impairment in at least one of these areas (Criterion D)					
A36[38]	Belgium, Colombia, France, Germany, Italy,	EUR, EMR, AMR	HIC, LMIC	NS	Community	2001-2003	Cross-sectional	Stratified multistage clustered probability sampling	Adult ADHD Clinical Diagnostic Scale (ACDS) V 1.2	DSM-IV, last 6 months	A clinical diagnosis of adult ADHD required six symptoms of either	Persistent adult ADHD	11422	388	18-44	NS

ID	Country	WHO	WB	Study setting	Origins of sample	Investigation Date	Study design	Sampling Strategy	Diagnostic tools for Adult ADHD	Criteria used for diagnosis	Definition of Adult ADHD	ADHD Category	Sample size	Cases	Age range	Female proportion
	Lebanon, Mexico, Netherlands, Spain, United States										inattention or hyperactivity–impulsivity during the 6 months before the interview (DSM–IV criterion A; American Psychiatric Associa-tion, 1994), at least two criterion A symptoms before age 7 years (criterion B), some impairment in at least two areas of living during the previous 6 months (criterion C) and clinically significant impairment in at least one					

ID	Country	WHO	WB	Study setting	Origins of sample	Investigation Date	Study design	Sampling Strategy	Diagnostic tools for Adult ADHD	Criteria used for diagnosis	Definition of Adult ADHD	ADHD Category	Sample size	Cases	Age range	Female proportion
											of these areas (criterion D). No attempt was made to operationalise DSM–IV diagnostic hierarchy rules (criterion E).					
A37[39]	USA	AMR	HIC	New England, mid-atlantic, east north central, west north central, south atlantic, east south central, west south central, mountain, pacific	Community	NS	Cross-sectional	Random sampling	NS	DSM-IV	We classified participants as positive for inattentive ADHD in childhood if six of the nine inattentive items were positive and less than six hyperactive-impulsive symptoms were positive. We classified participants as positive for hyperactive-impulsive ADHD if six of	Persistent adult ADHD	966	28	NS	0.52

ID	Country	WHO	WB	Study setting	Origins of sample	Investigation Date	Study design	Sampling Strategy	Diagnostic tools for Adult ADHD	Criteria used for diagnosis	Definition of Adult ADHD	ADHD Category	Sample size	Cases	Age range	Female proportion
											the nine hyperactive-impulsive items were positive and less than six inattentive symptoms were positive. We classified participants as combined type if six of the nine inattentive items were positive and six of the nine hyperactive-impulsive items were positive					
A38[40]	Netherlands	EUR	HIC	Nijmegen	Community	September 1997-March 1998	Cross-sectional	Probability sampling	ADHD DSM-IV rating scale	DSM-IV, last 6 months	The self-report questionnaire consisted of 26 items in total, with 23 items on current ADHD	Persistent adult ADHD	1813	45	18-75	0.553

ID	Country	WHO	WB	Study setting	Origins of sample	Investigation Date	Study design	Sampling Strategy	Diagnostic tools for Adult ADHD	Criteria used for diagnosis	Definition of Adult ADHD	ADHD Category	Sample size	Cases	Age range	Female proportion
											symptoms and three childhood items. A symptom was considered as present if the answer given to the item was 'often' or 'very often' (score of 2 or 3), using a cutoff of six and four current symptoms. For childhood symptoms a score 'often' or 'very often' on all three items was considered clinically relevant and taken as an index of the presence of ADHD in childhood					

Table S5. Quality scores for assessing the quality of the included articles (n=40)

ID	Author	Year Published	Quality score					
			Sample population	Sample size	Participation	Outcome assessment	Analytical methods	Total scores
A1[1]	Vigdis Elin Giaever Syrstada, et al.	2019	1	1	0	2	2	6
A2[2]	Kosuke Kajitani, et al.	2019	0	0	2	2	2	6
A3[3]	Paulo Mattos, et al.	2018	0	1	2	2	1	6
A4[4]	Flore Moulin, et al.	2018	2	1	0	2	2	7
A5[5]	Lucia Romo, et al.	2018	2	1	2	2	1	8
A6[6]	Meng Shi, et al.	2018	1	1	2	2	1	7
A7_a[7]	Etem Erdal Ersan	2017	1	1	2	2	1	7
A8[8]	John Fayyad, et al.	2017	1	1	0	2	2	6
A9[9]	Wakako Ito, et al.	2017	1	1	0	2	2	6
A10[10]	Manoj Kumar, et al.	2017	0	1	0	2	1	4
A11[11]	A.Stickleya, et al.	2017	2	1	0	2	2	7
A12[12]	E. S. Vitola, et al.	2017	2	1	2	2	2	9
A13[13]	ArthurCaye, et al.	2016	2	1	1	2	1	7
A14[14]	Filiz Özdemiroðlu, et al.	2016	0	0	0	2	1	3
A15[15]	Young-sook Kwak, et al.	2015	1	1	1	2	2	7
A16[16]	Terrie E. Moffitt, et al.	2015	2	1	2	2	2	9
A17[17]	P.MORTIER, et al.	2015	1	1	0	2	1	5
A18[1	EvelynVingilis, et al.	2015	2	1	0	2	2	7

ID	Author	Year Published	Quality score					
			Sample population	Sample size	Participation	Outcome assessment	Analytical methods	Total scores
8]								
A19[19]	Shahrokh Amiri, et al.	2014	1	1	0	2	1	5
A20[20]	Alexandre J. S. MORIN, et al.	2014	1	1	0	2	2	6
A21[21]	Estévez N, et al.	2014	2	1	1	2	2	8
A22[22]	Tuithof M, et al.	2014	2	1	0	2	2	7
A23[23]	Cortese S, et al.	2013	2	1	2	2	2	9
A24[24]	Jahangard L, et al.	2013	1	1	2	2	1	7
A25[25]	Ashor AW	2012	1	1	2	2	2	8
A26[26]	Das D, et al.	2012	2	1	0	2	2	7
A27[27]	de Zwaan M, et al.	2012	2	1	0	2	2	7
A7_b[28]	Kavakci O, et al.	2012	1	2	2	2	2	9
A28[29]	Michielsen M, et al.	2012	2	1	2	2	1	8
A29[30]	de Zwaan M, et al.	2011	2	1	0	2	2	7
A30[31]	Park S, et al.	2011	2	2	1	2	2	9
A31[32]	Soysal AS, et al.	2011	0	1	1	2	2	6
A32[33]	Atwoli L, et al.	2010	2	1	2	2	1	8

ID	Author	Year Published	Quality score					
			Sample population	Sample size	Participation	Outcome assessment	Analytical methods	Total scores
A33[34]	Garnier-Dykstra LM, et al.	2010	2	1	2	2	1	8
A34[35]	Polanczyk G, et al.	2010	2	2	0	2	2	8
A7_c[36]	Kilicoglu A, et al.	2009	1	1	0	2	1	5
A35[37]	Pagoto SL, et al.	2009	2	1	1	2	2	8
A36[38]	Fayyad J, et al.	2007	2	2	0	2	2	8
A37[39]	Faraone SV, et al.	2005	2	1	0	2	1	6
A38[40]	Kooij JJS, et al.	2005	2	1	2	2	2	9

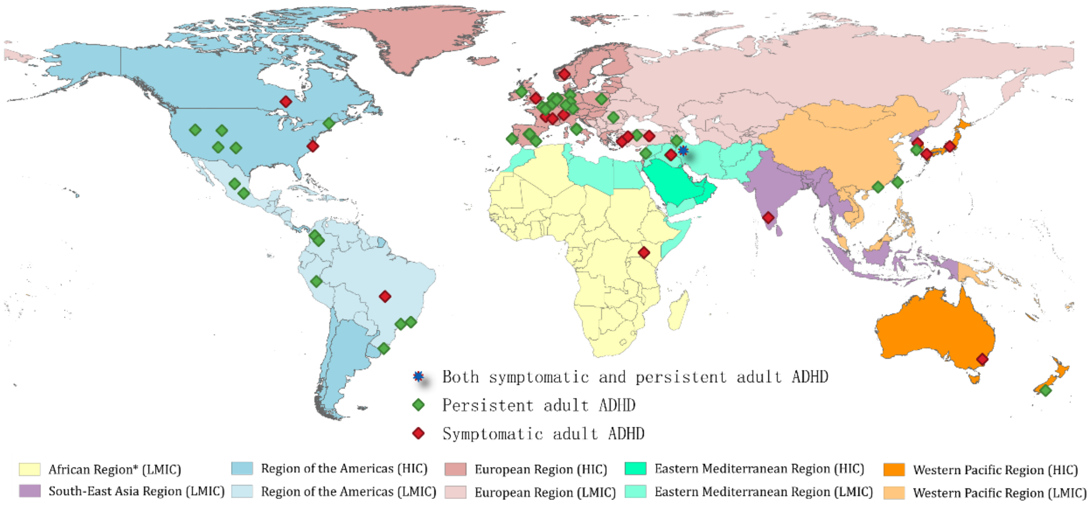

Figure S1. Location of the included articles reporting the prevalence of persistent adult ADHD and symptomatic adult ADHD

*Note: * Seychelles is a high-income country according to the latest World Bank income classification, but classified into the low-income and middle-income African Region due to its relatively small population size. The total number of articles that reported the prevalence of adult ADHD in the general population and corresponding sample size in each region were: 17 in the European Region (total sample: 36035), nine in the Region of Americas (total sample: 58442), seven in the Western Pacific Region (total sample: 22440), three in the Eastern Mediterranean Region (total sample: 1148), one in the South-East Region (India, total sample: 304), one in the African Region (Kenya, total sample:458). In addition, two articles were conducted across three regions (the European Region, the Eastern Mediterranean Region, and the Region of Americas, total sample: 38166).*

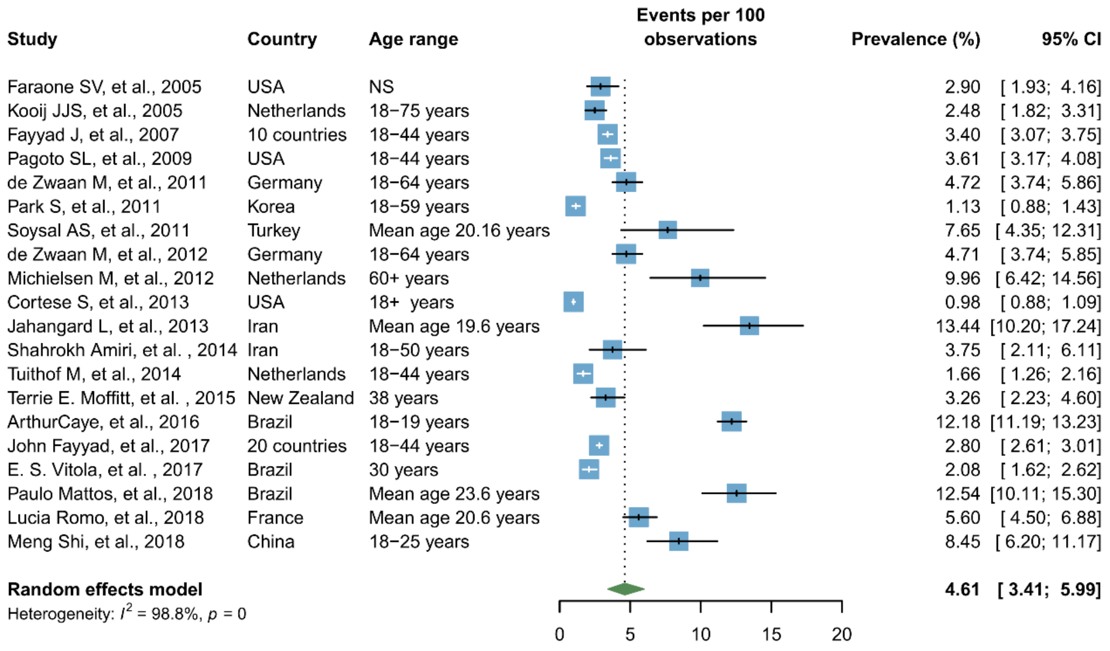

Figure S2. Pooled prevalence of persistent adult ADHD (n=20)

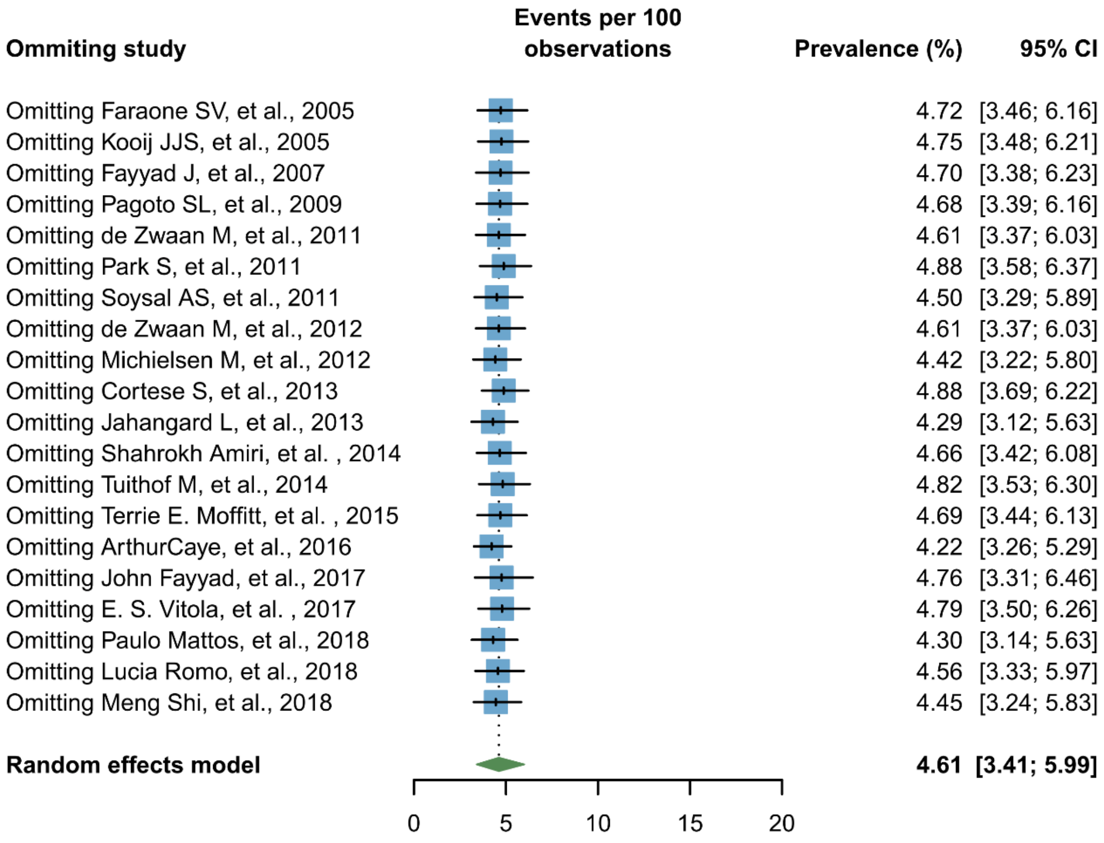

Figure S3. Leave-one-out sensitivity analysis of the influence of single study on the pooled prevalence of persistent adult ADHD

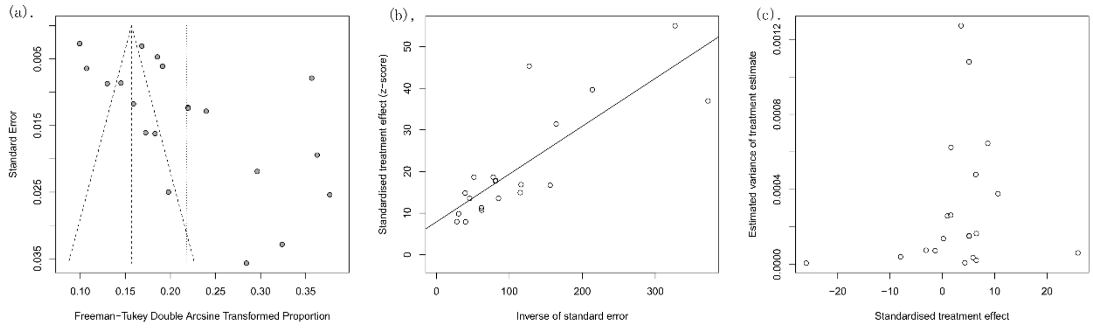

Figure S4. Publication bias of studies on the prevalence of persistent adult ADHD

Note: (A) Funnel plot; (B) Egger's test; (C) Begg's test.

Study	Country	Age range	Prevalence (%)	95% CI
Kilicoglu A, et al., 2009	Turkey	Mean age 18.97 years	15.55	[13.98; 17.23]
Atwoli L, et al., 2010	Kenya	17−46 years	9.17	[6.69; 12.19]
Garnier−Dykstra LM, et al., 2010	USA	21−23 years	10.00	[8.28; 11.95]
Polanczyk G, et al., 2010	Brazil	18+ years	5.79	[4.98; 6.68]
Kavakci O, et al., 2012	Turkey	17−44 years	4.90	[3.63; 6.44]
Ashor AW, 2012	Iraq	18−25 years	16.62	[12.93; 20.87]
Das D, et al., 2012	Australia	47−54 years	6.17	[5.17; 7.28]
Jahangard L, et al., 2013	Iran	Mean age 19.6 years	16.54	[12.97; 20.62]
Alexandre J. S. MORIN, et al. , 2014	France	20−64 years	11.19	[9.44; 13.13]
Estevez N, et al., 2014	Switzerland	17−28 years	4.00	[3.50; 4.54]
Young−sook Kwak, et al. , 2015	Korea	18+ years	7.55	[6.47; 8.74]
P.MORTIER, et al. , 2015	Belgium	18+ years	5.39	[4.77; 6.05]
EvelynVingilis, et al. , 2015	Canada	18+ years	3.39	[2.84; 4.00]
Filiz Ozdemiro?lu, et al. , 2016	Turkey	Mean age 20.42 years	7.97	[5.90; 10.49]
Etem Erdal Ersan, 2017	Turkey	18−35 years	3.13	[2.23; 4.25]
Wakako Ito, et al. , 2017	Japan	20−69 years	6.13	[5.66; 6.62]
Manoj Kumar, et al. , 2017	India	19+ years	25.66	[20.84; 30.95]
A.Stickleya, et al. , 2017	United Kingdom	18−95 years	5.32	[4.82; 5.87]
Flore Moulin, et al., 2018	France	18−37 years	7.08	[5.71; 8.68]
Vigdis Elin Giaever Syrstada, et al., 2019	Norway	18−40 years	10.87	[8.65; 13.43]
Kosuke Kajitani, et al., 2019	Japan	Mean age 21.4 years	22.35	[19.51; 25.39]
**Random effects model**			**8.83**	**[7.23; 10.57]**

Heterogeneity: $I^2 = 97.6\%$, $p < 0.01$

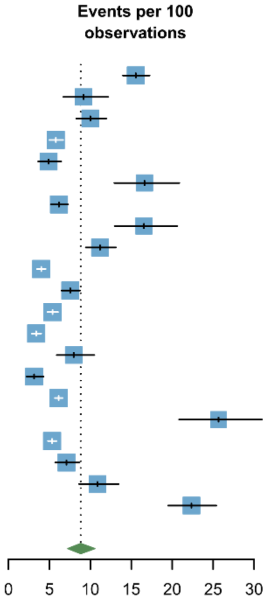

Figure S5. Pooled prevalence of symptomatic adult ADHD (n=21)

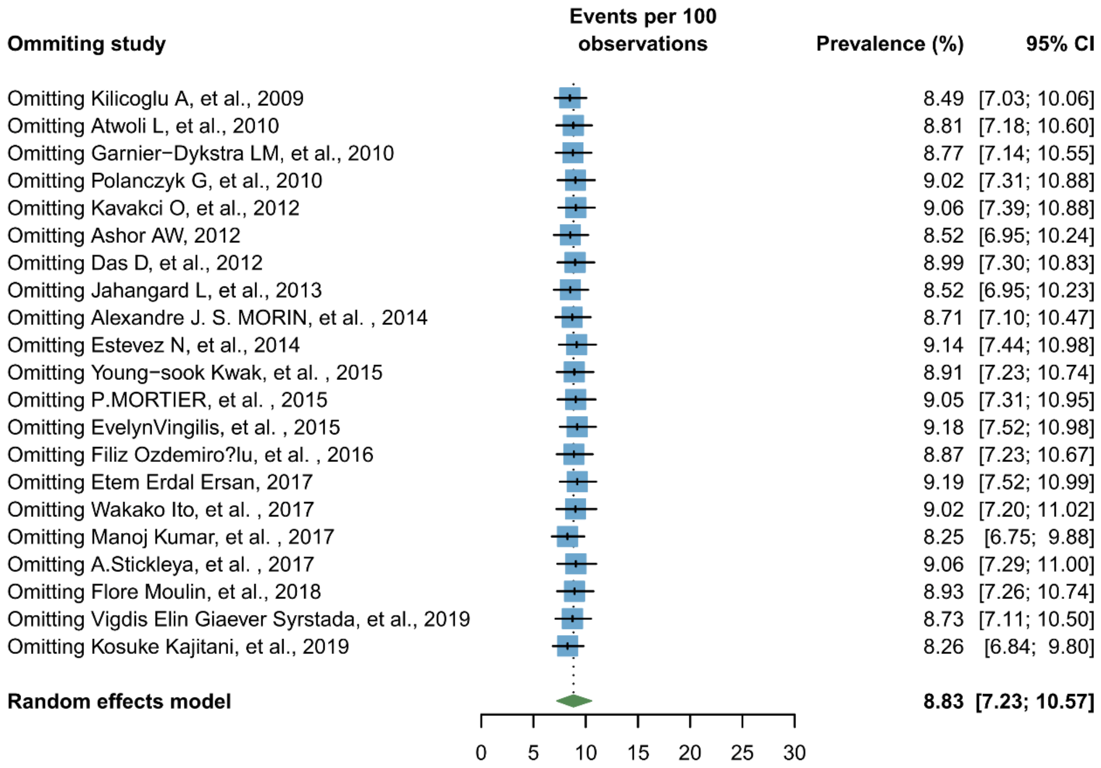

Figure S6. Leave-one-out sensitivity analysis of the influence of single study on the pooled prevalence of symptomatic adult ADHD

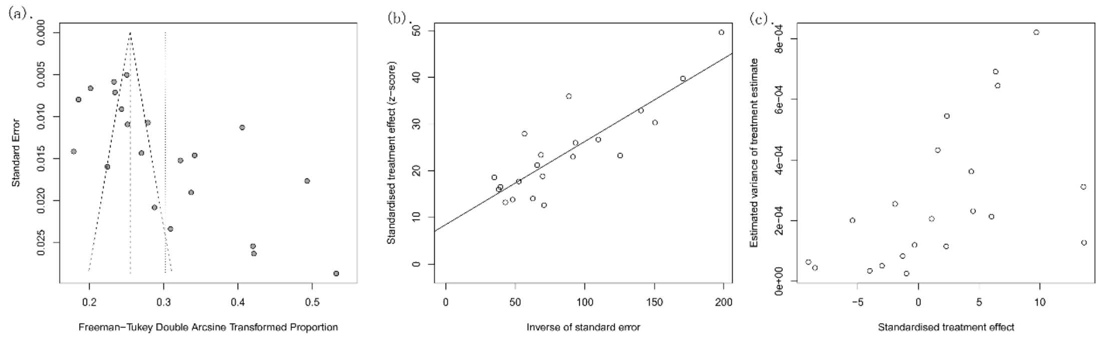

Figure S7. Publication bias of studies on the prevalence of symptomatic adult ADHD

Note: (A) Funnel plot; (B) Egger's test; (C) Begg's test.

References

1. Syrstad, V.E.G., et al., *Cyclothymic temperament: Associations with ADHD, other psychopathology, and medical morbidity in the general population.* Journal of Affective Disorders, 2020. **260**: p. 440-447.
2. Kajitani, K., et al., *Relevance of hoarding behavior and the traits of developmental disorders among university students: A self-reported assessment study.* BioPsychoSocial Medicine, 2019. **13**.
3. Mattos, P., B.P. Nazar, and R. Tannock, *By the book: ADHD prevalence in medical students varies with analogous methods of addressing DSM items.* Revista brasileira de psiquiatria (Sao Paulo, Brazil : 1999), 2018. **40**(4): p. 382-387.
4. Moulin, F., et al., *Prevalence and Psychosocial Correlates of ADHD Symptoms in Young Adulthood: A French Population-Based Study.* Journal of attention disorders, 2018. **22**(2): p. 167-181.
5. Romo, L., et al., *Attention-deficit hyperactivity disorder and addictions (substance and behavioral): Prevalence and characteristics in a multicenter study in France.* Journal of behavioral addictions, 2018. **7**(3): p. 743-751.
6. Shi, M., et al., *Associations between symptoms of attention-deficit/ hyperactivity disorder and life satisfaction in medical students: the mediating effect of resilience.* BMC medical education, 2018. **18**(1): p. 164-164.
7. Ersan, E.E., *Signs of adult ADHD in university students and related factors.* Anadolu Psikiyatri Dergisi, 2017. **18**(4): p. 353-361.
8. Fayyad, J., et al., *The descriptive epidemiology of DSM-IV Adult ADHD in the World Health Organization World Mental Health Surveys.* Attention deficit and hyperactivity disorders, 2017. **9**(1): p. 47-65.
9. Ito, W., et al., *Excessive daytime sleepiness in adults with possible attention deficit/hyperactivity disorder (ADHD): a web-based cross-sectional study.* Sleep medicine, 2017. **32**: p. 4-9.
10. Kumar, M., et al., *Association of attention-deficit hyperkinetic disorder with alcohol use disorders in fishermen.* Journal of Neurosciences in Rural Practice, 2017. **8**(5 Supplement 1): p. 78-82.
11. Stickley, A., et al., *Attention-deficit/hyperactivity disorder and physical multimorbidity: A population-based study.* European psychiatry : the journal of the Association of European Psychiatrists, 2017. **45**: p. 227-234.
12. Vitola, E.S., et al., *Exploring DSM-5 ADHD criteria beyond young adulthood: phenomenology, psychometric properties and prevalence in a large three-decade birth cohort.* Psychological medicine, 2017. **47**(4): p. 744-754.
13. Caye, A., et al., *Attention-Deficit/Hyperactivity Disorder Trajectories From Childhood to Young Adulthood: Evidence From a Birth Cohort Supporting a Late-Onset Syndrome.* JAMA psychiatry, 2016. **73**(7): p. 705-712.
14. Ozdemiroglu, F., et al., *Sociodemographic and clinical characteristics in medical students with attention deficit hyperactivity disorder.* Tip Fakultesi Ogrencilerinde Dikkat Eksikligi ve Hiperaktivite Bozuklugunun Sosyodemografik ve Klinik Ozellikleri., 2016. **19**(1): p. 5-14.
15. Kwak, Y.S., Y.E. Jung, and M.D. Kim, *Prevalence and correlates of attention-deficit hyperactivity disorder symptoms in korean college students.* Neuropsychiatric Disease and Treatment, 2015. **11**: p. 797-802.
16. Moffitt, T.E., et al., *Is Adult ADHD a Childhood-Onset Neurodevelopmental Disorder? Evidence From a Four-Decade Longitudinal Cohort Study.* The American journal of psychiatry, 2015. **172**(10): p. 967-977.
17. Mortier, P., et al., *The epidemiology of ADHD in first-year university students.* Tijdschrift voor psychiatrie, 2015. **57**(9): p. 635-644.
18. Vingilis, E., et al., *Attention Deficit Hyperactivity Disorder Symptoms, Comorbidities, Substance Use, and Social Outcomes among Men and Women in a*

Canadian Sample. BioMed research international, 2015. **2015**: p. 982072-982072.
19. Amiri, S., et al., *Prevalence of adult attention deficit hyperactivity disorder (adult ADHD): Tabriz.* Iranian Journal of Psychiatry, 2014. **9**(2): p. 83-88.
20. Caci, H.M., A.J.S. Morin, and A. Tran, *Prevalence and correlates of attention deficit hyperactivity disorder in adults from a French community sample.* The Journal of nervous and mental disease, 2014. **202**(4): p. 324-332.
21. Estévez, N., et al., *Prevalence of and associated factors for adult attention deficit hyperactivity disorder in young Swiss men.* PloS one, 2014. **9**(2): p. e89298-e89298.
22. Tuithof, M., et al., *Prevalence, persistency and consequences of ADHD in the Dutch adult population.* Tijdschrift voor psychiatrie, 2014. **56**(1): p. 10-19.
23. Cortese, S., et al., *Adult attention-deficit hyperactivity disorder and obesity: epidemiological study.* The British journal of psychiatry : the journal of mental science, 2013. **203**(1): p. 24-34.
24. Jahangard, L., et al., *Among a sample of Iranian students, adult attention deficit hyperactivity disorder is related to childhood ADHD, but not to age, gender, socioeconomic status, or birth order--an exploratory study.* International journal of psychiatry in clinical practice, 2013. **17**(4): p. 273-278.
25. Ashor, A.W., *Variable influence of the degree of smoking dependence on adult attention deficit/hyperactivity disorder in Iraqi medical students.* Neurosciences (Riyadh, Saudi Arabia), 2012. **17**(3): p. 241-7.
26. Das, D., et al., *A population-based study of attention deficit/hyperactivity disorder symptoms and associated impairment in middle-aged adults.* PloS one, 2012. **7**(2): p. e31500.
27. de Zwaan, M., et al., *The estimated prevalence and correlates of adult ADHD in a German community sample.* European archives of psychiatry and clinical neuroscience, 2012. **262**(1): p. 79-86.
28. Kavakci, O., et al., *Prevalence of attention-deficit/hyperactivity disorder and co-morbid disorders among students of Cumhuriyet University.* European Journal of Psychiatry, 2012. **26**(2): p. 107-117.
29. Michielsen, M., et al., *Prevalence of attention-deficit hyperactivity disorder in older adults in The Netherlands.* The British journal of psychiatry : the journal of mental science, 2012. **201**(4): p. 298-305.
30. de Zwaan, M., et al., *Association between obesity and adult attention-deficit/hyperactivity disorder in a German community-based sample.* Obesity facts, 2011. **4**(3): p. 204-211.
31. Park, S., et al., *Prevalence, correlates, and comorbidities of adult ADHD symptoms in Korea: results of the Korean epidemiologic catchment area study.* Psychiatry research, 2011. **186**(2-3): p. 378-383.
32. Soysal, A.S., et al., *The prevalence of attention deficit hyperactivity disorder symptoms among nursing students.* Hemsirelik ogrencilerinde dikkat eksikligi hiperaktivite bozuklugu belirtilerinin yayginligi., 2011. **49**(1): p. 27-34.
33. Atwoli, L., et al., *Self-reported attention deficit and hyperactivity disorder symptoms among university students in Eldoret, Kenya.* East African medical journal, 2010. **87**(5): p. 187-191.
34. Garnier-Dykstra, L.M., et al., *Self-reported adult attention-deficit/hyperactivity disorder symptoms among college students.* Journal of American college health : J of ACH, 2010. **59**(2): p. 133-136.
35. Polanczyk, G., et al., *ADHD in a representative sample of the Brazilian population: estimated prevalence and comparative adequacy of criteria between adolescents and adults according to the item response theory.* International journal of methods in psychiatric research, 2010. **19**(3): p. 177-184.

36. Kilicoglu, A., et al., *The assessment of attention deficit hyperactivity disorder symptoms in university students.* Universite ogrencilerinde kendilerinin bildirdikleri dikkat eksikligi hiperaktivite bozuklugu belirtilerinin degerlendirilmesi., 2009. **10**(2): p. 88-93.
37. Pagoto, S.L., et al., *Association between adult attention deficit/hyperactivity disorder and obesity in the US population.* Obesity (Silver Spring, Md.), 2009. **17**(3): p. 539-544.
38. Fayyad, J., et al., *Cross-national prevalence and correlates of adult attention-deficit hyperactivity disorder.* The British journal of psychiatry : the journal of mental science, 2007. **190**: p. 402-409.
39. Faraone, S.V. and J. Biederman, *What is the prevalence of adult ADHD? Results of a population screen of 966 adults.* Journal of attention disorders, 2005. **9**(2): p. 384-391.
40. Kooij, J.J.S., et al., *Internal and external validity of attention-deficit hyperactivity disorder in a population-based sample of adults.* Psychological medicine, 2005. **35**(6): p. 817-827.
